# Field evidence of UK wild bird exposure to fludioxonil and extrapolation to other pesticides used as seed treatments

**DOI:** 10.1007/s11356-021-17097-y

**Published:** 2021-11-15

**Authors:** Cannelle Tassin de Montaigu, Dave Goulson

**Affiliations:** School of Life Sciences, University of Sussex, Falmer, East Sussex UK

**Keywords:** Pesticide, Seed treatment, Bird, Farmland, Fungicide, LD50

## Abstract

We determine the exposure of wild birds to pesticides via consumption of fludioxonil-treated winter wheat seeds following autumn drilling. We recorded the density of seeds left on the soil surface, bird density, and consumption of pesticide-treated seed by birds using camera traps. We calculated the dose ingested by each bird species in a single feeding bout and if they ate treated seeds exclusively for 1 day. We extrapolated this for an additional 19 pesticides commonly used as seed treatments, assuming equal consumption rates. All three fields contained grains on the soil surface (mean 7.14 seeds/m^2^ on sowing day). In total, 1,374 granivorous birds spanning 18 different species were observed in the fields, with 11 species filmed eating the seeds. Fludioxonil appears to pose a low risk to birds, with <1.14% of the LD50 potentially ingested by a bird for a daily maximum amount of seeds. Analysis of the further 19 pesticides commonly used as seed dressings suggests that the neonicotinoid insecticides imidacloprid, clothianidin, and thiamethoxam represent the highest risk for granivorous birds. For example, chaffinch (*Fringilla coelebs*) could consume 63% of LD50 of imidacloprid in a single feeding bout, and 370% in a day. Further investigation is clearly required to determine whether seeds treated with these other pesticides are consumed as readily as those treated with fludioxonil, as if so this is likely to cause significant harm.

**Supplementary Information:**

The online version contains supplementary material available at 10.1007/s11356-021-17097-y.

## Introduction

The application of pesticides on crops has been perceived as one of the drivers of the decline of farmland bird populations (Chamberlain 2002; Hallmann et al. 2014). Pesticide usage can negatively affect avian population through direct toxicity; altering survival, health, and/or reproduction; and indirect pathways such as food reduction and habitat degradation and loss (Geiger et al. 2010; Hallmann et al. 2014; Potts and Aebischer 1995; Wilson et al. 1999; Watkinson et al. 2000).

Pesticide-coated seeds provide a convenient method for pesticide application on crops as they decrease the need to spray, reduce the exposure to the farmer, deposit the active substance on a smaller area, and, in theory at least, decrease the risk posed to non-target species (Dewar and Asher 1994; Hart and Clook 1994). However, some seeds are not buried during sowing, remain on the soil surface, and are thus available to granivorous vertebrates (Goulson 2013). De Snoo and Luttik (2004) found that the type of crop, soil condition, sowing technique, location on the field, and season all influence greatly the percentage of seeds that remain available on the soil surface, which is typically between 0.5 and 9.2%. They found an important difference between the autumn and spring, with the abundance of seeds on the surface in autumn higher by a factor of 13 (probably due to harder soil conditions), and 3.5 times higher on the headlands than in the centre of fields.

When winter cereal sowing became common, winter cereal crops became a key component in the diet of many granivorous birds (Browne and Aebischer 2003; Robinson 2004; Perkins et al. 2007). For example, the digestive contents of red-legged partridge (*Alectoris rufa*) were analysed during the cereal sowing season in Spain and Lopez-Antia et al. (2016) found that cereal seeds represented up to 89.3% of the consumed biomass. Since seeds are commonly coated with pesticides, this provides a route of exposure of birds (Lopez-Antia et al. 2016; Holland et al. 2006). Numerous studies have shown exposure or poisoning incidents involving a variety of bird species consuming seeds treated with neonicotinoids, pyrethroids, organochlorines, organophosphates, or carbamates (Murton and Visozo 1963; Porter 1977; De Snoo et al. 1999; Millot et al. 2017; Corcellas et al. 2017; MacDonald et al. 2018; Lennon et al. 2020b).

The dose ingested by birds in a single feeding bout on treated seeds can be sufficient to cause lethal and sublethal effects (Prosser and Hart 2005). Even if less acutely toxic to avian species, fungicides are applied in greater quantities than insecticides (Tassin de Montaigu and Goulson 2020) and could represent a risk to birds. Mateo et al. (2016) estimated, using daily food intake, that the fungicides thiram and tebuconazole could represent a risk to red-breasted geese (*Branta ruficollis*). Experimental studies have shown that exposure to triazole fungicides used in seed coating treatments can affect reproduction in Japanese quail (*Coturnix japonica*) by disturbing testicular histology and sperm production (Grote et al. 2008). Similarly, exposure to seeds treated with the fungicide difenoconazole was found to reduce the reproductive success of the red-legged partridge by reducing the fertility rate of eggs (Lopez-Antia et al. 2013). More recently, Lopez-Antia et al. (2018) also found a 56–62% brood size reduction in partridge feeding on seeds treated with the fungicide flutriafol, even when doses were below the recommended application rates.

Although consumption of treated seeds would seem to be a source of significant exposure of granivorous birds to a range of different pesticides, our knowledge on wild bird exposure to pesticides via this route is still sparse. We have limited data as to which species consume treated seeds, and on their consumption rates especially when it comes to pesticides other than insecticides (Prosser and Hart 2005; Lopez-Antia et al. 2016; Millot et al. 2017; Lennon et al. 2020a). This study aims to investigate which bird species feeds on pesticide-coated wheat seeds during winter cereal sowing season in the UK, and to quantify the exposure of different wild bird species to seeds coated with the fungicide fludioxonil. Additionally, we extrapolated these findings to an additional 19 pesticides that are commonly used as seed treatment worldwide, to obtain a more general understanding of the potential risks to birds associated with current seed treatments.

## Material and methods

Methods were based on a previous study by Lennon et al. (2020a) which focussed on exposure to clothianidin, a neonicotinoid insecticide, during the autumn sowing season. We collected data from three fields on two farms located in East Sussex, UK, during the autumn sowing seasons of 2020 (Hartfield: 51.1023° N, 0.1115° E; Barnham: 50.8278° N, 0.6357° W). The fields were sown with fludioxonil-dressed wheat seeds (respectively Beret Gold® and Vibrance DUO®; Syngenta, UK; max application rate of 2L/tonne of seed). Farmers continued their standard practice, seed coating preparation, and sowing methods throughout the season and they were aware that researchers would be surveying their fields. Table 1 gives full details on the farms and field characteristics. All data collection was conducted by the same observer.

**Table 1:** General information about the study sites

Farm	Field	Area (ha)	Soil type	Tillage (cm)	Sowing			% hedgerow	Adjacent habitat	Chemicals used before/during study
					Date (D/M/Y)	Rate (kg/ha)	Technique			
Barnham	1	17.86	Sandy clay/brick earth	Ploughed to approx. 25cm immediately before the power harrow and drill combination	13/10/2020	168	Drilled after plough and power harrow	3.92	Farmland	NA
Hartfield	1	4.00	Tunbridge Wells sand	None	23/09/2020	165	Direct drilled	0.34	Farmland and woodland	Glyphosate, flufenacet, flurtamone and diflufenican (Movon)
Hartfield	2	2.00	Tomotley sand	None	25/09/2020	165	Direct drilled	0.50	Farmland and woodland	Glyphosate, flufenacet, flurtamone and diflufenican (Movon)

### Surface seed density and seed cluster counts

On days 0, 1, 3, 6, 9, and 12 (day 0 being within 24h of drilling), the number of treated wheat seeds visible on the ground surface, along transects bisecting the headlands and field centre, was recorded in 60 quadrats (0.25 m^2^), 20 quadrats in the field centre and 20 quadrats at each of the two field headlands, at least 5m apart (spacing of the quadrats was bigger on larger fields). In addition, on the day that seeds were drilled (day 0) in each field, the observer walked along the field boundary and counted seed clusters, defined as a spillage if there were >10 seeds within a 0.25-m^2^ area. Clusters of >100 seeds were also noted (Table 1).

### Surveys of bird abundance and bird density

On days 0, 1, 3, 6, 9, and 12, bird abundance was measured in two manners:

First, when arriving on site, a scan of all species present on the entire field was completed by using binoculars. Second, a flush count was conducted whilst walking field transects (a maximum of three transects per field separated by at least 100m, following Lennon et al. 2020a). The location of each bird was recorded (field boundaries, centre, or both) and, when possible, species were identified. For statistical analysis, the abundance of only seed-eating birds was used, which excluded the common buzzard (*Buteo buteo*), house martins (*Delichon urbicum*), kittiwakes (*Rissa tridactyla*), and herring gulls (*Larus argentatus*). Additionally, for statistical analysis, bird density (birds/ha) for each of the fields was used.

### Camera trapping to quantify seed consumption

Two Camera traps (Bushnell Natureview Cam Essential HD, USA) were installed on day 0 in each field. One camera was placed in the centre of the field and the second on the field boundary. A maximum of 200g of seeds (approx. 3,000 seeds) obtained from the farmer were placed approximately 2m in front of the camera. The cameras remained active until seeds were depleted or for no longer than 20 days. Cameras recorded for 10 continuous seconds when activated by movement. The time spent in front of the camera and the number of treated seeds ingested (or seed intake) by each individual were counted. Treated seeds were commercially coloured with a bright red dye and thus easily recognised in the footage. Two consecutive feeding bouts by birds of the same species were counted as two different individuals except when the interval was less than 5s and the observer was confident that it was the same individual, based on its position in the field of view. This approach will tend to lead to a conservative estimate of seed consumption per bird.

### Estimation of the percentage of LD50 ingested

Firstly, we estimated the LD50 (mg/bird) (the dose of substance that gives a 50% probability of death), for each species observed feeding on treated seeds using scaling factors based on weight (Mineau et al. 1996, 2001; Tassin de Montaigu and Goulson 2020). For the five insecticides and 15 fungicides that are frequently used as seed treatment (in Europe, North America, and/or Asia), the LD50 (mg/kg of body weight) was obtained from the Pesticides Properties DataBase (PPDB University of Hertfordshire). The maximum application rates recommended by manufacturers were available on the seed treatment labels. Note that some of these chemicals, such as neonicotinoid insecticides, are now banned from use as seed dressings in Europe but are widely used elsewhere in the world, and even within Europe, individual countries often grant temporary derogations to allow their use as seed dressings.

Secondly, we calculated the quantity of pesticide coating on seeds and the corresponding quantity ingested by species according to their maximum seed intake (maximum number of seeds consumed per species) in a feeding bout. For instance, both Beret gold® and Vibrance DUO® had a concentration of fludioxonil of 25 g/L and the labels recommended a maximum dose of 2 L/tonne of seed. This corresponds to a maximum of 50 g fludioxonil/tonne of seed or 0.05 g of fludioxonil/kg of seed. As an example, we observed ring-necked pheasants (*Phasianus colchicus*) consumed a maximum of 301 seeds in a foraging bout. The average weight of one wheat seed was taken to be 0.045 g (Bouaziz and Hicks 1990). Therefore, the maximum weight of seeds consumed for the pheasant is 13.5 g. We can then calculate the quantity of fludioxonil consumed by the pheasant for its maximum intake, which is 0.677 mg for 301 seeds.

Thirdly, we calculated the corresponding percentage of LD50 consumed by the birds. For example, the fludioxonil LD50 for pheasant is about 2,680 mg/bird; the mass of fludioxonil consumed by the pheasant in 301 seeds being 0.677 mg, the corresponding percentage of LD50 consumed by pheasants for 301 seeds is 0.025%.

Furthermore, Crocker et al. (2002) found a method estimating the mean daily food intake (g) for different food types (e.g. cereal, arthropods, dicot crop leaves) for various bird and mammal species based on daily energy expenditure. We used the mean food intake they found for ‘cereal’ and ‘weed seed’ for some of the species we observed, and we similarly calculated what a total daily intake of treated seed would represent as a percentage of LD50 consumed.

Since all species swallowed the seeds whole except the common chaffinch (*Fringilla coelebs*) which de-husked seeds, we assumed a worst-case scenario with no de-husking and maximum exposure of the seed dressing.

### Statistical analysis

All analyses were performed using R v. 4.0.3 (R Core Team 2020). We used general linear models (GLMs) using the package ‘lme4’ (Bates et al. 2015) with significance level set at 0.05, with temperature (°C), cloud cover (%), rainfall (mm), and wind (km/h) as random factors, and field as a fixed factor to investigate the relationship between surface seed density, days post-sowing, location on field (headland or centre), and bird density. Except for surface seed density where a Gaussian distribution was used, all GLMs were run using a negative binomial distribution to account for overdispersion. Three separate models were fitted, (a) seed density (seeds/m^2^) as a function of days post-sowing, the mean surface seed density was calculated per field; (b) bird density (birds/ha) as a function of days post-sowing; (c) bird density as a function of seed density. We also compared the average seed consumption (number of seeds consumed per feeding bout) according to camera location on the fields (centre field or boundary).

## Results

### Seed and bird density

A total of 60 clusters of over 10 seeds and 14 clusters of over 100 seeds were counted along the field boundaries of Barnham farm, 69 clusters of 10 seeds and 11 clusters of over 100 seeds were counted along the field boundaries of Hartfield field 1, and 30 clusters over 10 seeds and 5 clusters over 100 seeds were counted along the field boundaries of Hartfield field 2. This represents for Barnham farm a minimum of 2000 seeds, for Hatfield field 1 a minimum of 1790 seeds, and for Hartfield field 2 a minimum of 850 seeds available on the soil surface just from those clusters.

**Fig. 1:**
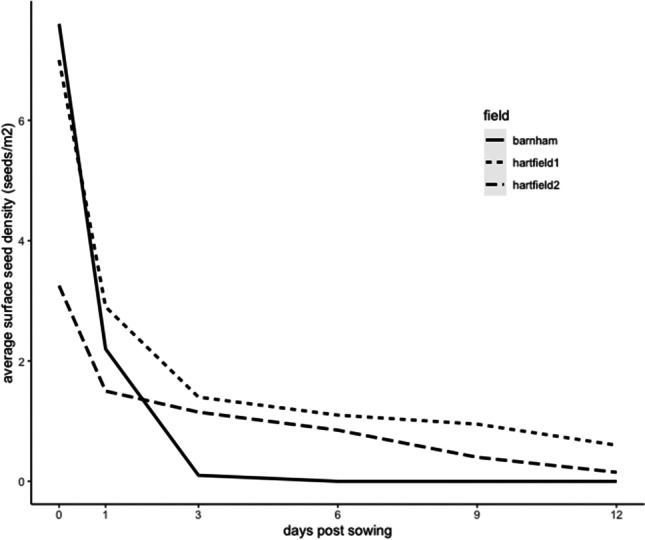
Seed density according to the days post-sowing for the different fields

Over all fields, the seed density ranged from 0 to 124 seeds/m^2^ with an average surface seed density of 7.14 seeds/m^2^ on day 0, more specifically 9.7 seeds/m^2^ on Barnham farm and 5.87 seeds/m^2^ for Hartfield farm. This average surface seed density significantly decreased on all fields over time (*t*_35_= −3.87, *p*<0.001). After 4 days (from day 0 to day 3 post-sowing), Barnham farm average surface seed density had decreased by 98.6%, Hartfield field 1 decreased by 77.61%, and Hartfield field 2 decreased by 66.75% (Fig. 1).

**Fig. 2:**
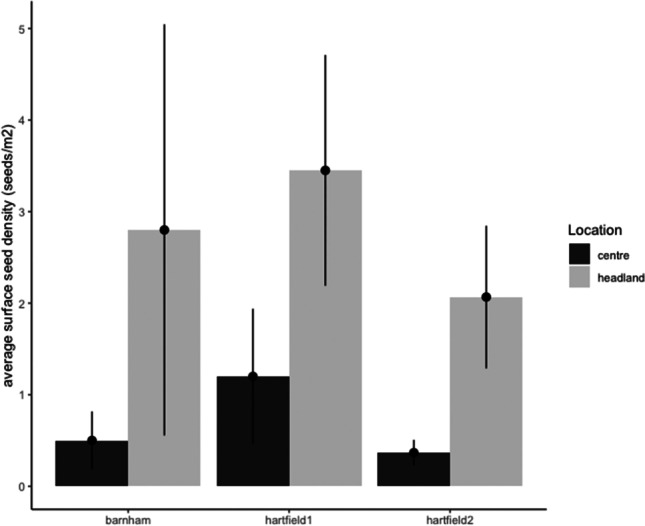
Seed density according to location on field for all fields

The average surface seed density on headlands (2.77 seeds/m^2^) was 4 times higher than the average surface seed density found in the centre of fields (0.68 seeds/m^2^) for all days post-sowing (*t*_35_=3.045, *p*<0.01; Fig. 2).

**Fig. 3:**
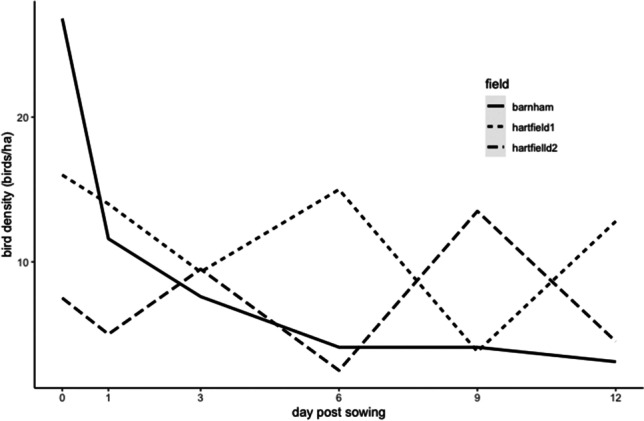
Total bird density (birds per hectare) according to day post-sowing for the different fields

**Fig. 4:**
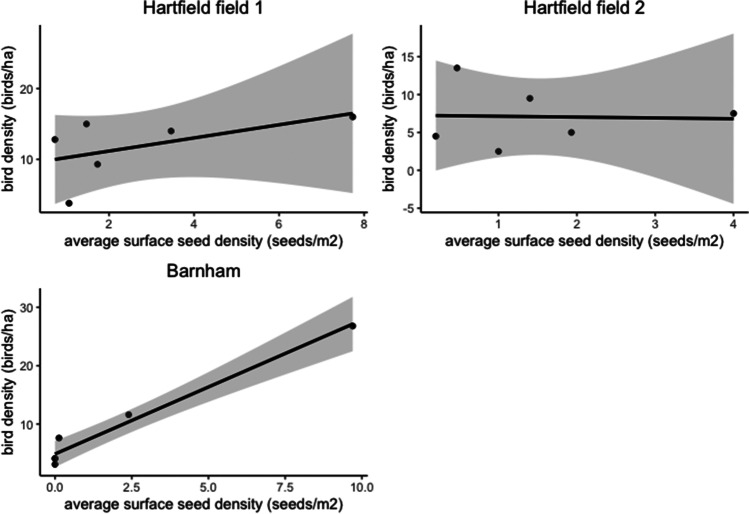
Bird density (birds per hectare) according to seed density (seeds per square metre) for the different fields. The shaded band shows the 95% confidence interval on the fitted values

Across all fields, we observed a total of 1,374 individual seed-eating birds distributed across 18 species, plus 88 unidentified individuals (too far away or too fast for the observer to be certain of the species; supplementary material, Table 1). For the different bird abundance recordings, a mean count over all fields of 11.2 granivorous birds on arrival and 8.13 granivorous birds along transects suggested that the presence of the observer lowered the number of birds present on site. Barnham field showed the highest seed-eating bird abundance with 1,006 birds observed during the study, compared to 283 birds for Hartfield field 1 and 85 birds for Hartfield field 2. In terms of density of birds, this represents 560 birds/ha for Barnham field, compared to 70.8 birds/ha for Hartfield field 1 and 42.5 birds/ha for Hartfield field 2. The bird density (granivorous birds per hectare) tended to decrease with days post-sowing (*t*_17_= −0.996, *p*=0.34), but this was explained only by Barnham farm for which the total bird density significantly dropped by 88.4% between day 0 and day 12 post-sowing (*t*_5_= −4.22, *p*=0.01; Fig. 3). Similarly, a positive relationship between average surface seed density and bird density was much stronger at Barnham (*t*_5_=4.130, *p*=0.01) than across all three fields (*t*_17_=1.957, *p*=0.07; Fig. 4).

### Seed consumption

On the camera footage, we observed 11 different bird species eating the wheat seeds across the three fields (supplementary material, Table 1). The carrion crow (*Corvus corone*), common chaffinch, ring-necked pheasant, red-legged partridge, and rook (*Corvus frugilegus*) were the five bird species observed most. Between them, carrion crow and common chaffinch comprised more than half of all recordings (27.8% and 22.8% of observations, respectively) although both were observed exclusively on Barnham farm. The seed consumption recorded per observation ranged from 1 to 405 seeds with an average across species of 21.1 seeds. The bird that consumed the most seeds per feeding bout was a feral pigeon (*Columba livia domesticus*) with 405 seeds consumed in 5 min and 30 s, corresponding to 10.9% of the species’ average body weight. The highest average consumption rate of seeds happened on the boundary cameras, and some species were only observed on field boundaries, for example the Eurasian magpie (*Pica pica*) and the feral pigeon.

### Estimation of the percentage of LD50 ingested

Of the 20 most used pesticides as seed treatments, the insecticides imidacloprid, clothianidin, and thiamethoxam present the highest risk of birds, based on the percentage of LD50 ingested in a single feeding bout (63.23%, 6.44%, and 4.33% for the chaffinch and 56.90%, 5.80%, and 3.89% for the feral pigeon, respectively, Table 2). The fungicide carboxin and the insecticide tefluthrin also appear to pose some risk, providing 3.61% and 3.91% of the LD50 for chaffinch and 3.25% and 3.51% for the feral pigeon, respectively. Fludioxonil, which was the active ingredient used as the seed treatment on both farms, represented 0.19% of the LD50 ingested by chaffinch and 0.17% of the LD50 ingested by feral pigeon. The rest of the pesticides listed in our analysis appear to provide little to no threat to birds via this source of exposure.

**Table 2:**
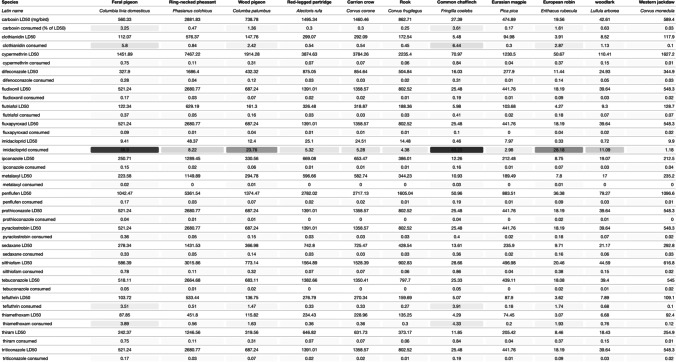
LD50 and percentage of LD50 consumed by the 11 species from our camera trap study in a single feeding bout, for the 20 most used pesticide seed treatments. The dark to light grey gradient indicates the highest to lowest percentage of LD50 consumed

As one might expect, the patterns are broadly similar if we examine the doses consumed if we assume that birds fed only treated seeds all day. Imidacloprid (370%), clothianidin (37.8%), and thiamethoxam (25.3%) again presented the highest percentage of LD50 ingested for chaffinch, followed by carboxin (21.2%) and tefluthrin (22.9%, Table 3).

**Table 3:**
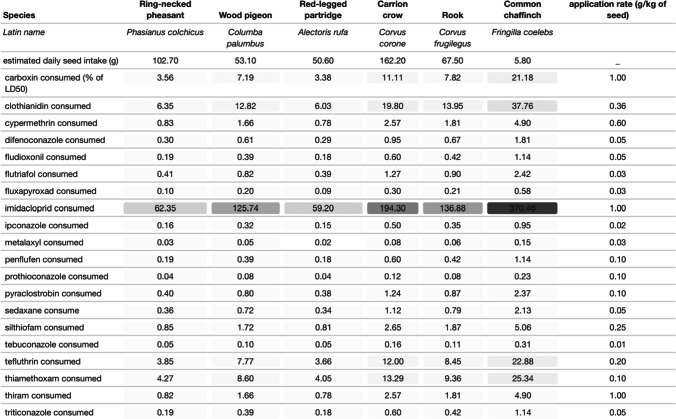
Percentage of LD50 consumed by species if pesticide-coated seeds correspond to the daily intake, for the 20 most used pesticide seed treatments. The maximum recommended application rate (g/kg of seeds) by the companies is provided in the last column. The dark to light grey gradient indicates the highest to lowest percentage of LD50 consumed. Only 6 out of our 11 species were available for the estimated daily food intake calculated by Crocker et al. (2002)

Comparing bird species in our study, chaffinch appears to be the species most likely to receive a harmful dose of pesticide from seed-treated grain, followed by feral pigeon, European robin (*Erithacus rubecula)*, wood pigeon (*Columba palumbus*), and woodlark (*Lullula arborea*; Table 2). When looking at the species occurrence between field observation and camera footage, we found that some species, such as skylarks (*Alauda arvensis*) or house sparrows (*Passer domesticus*), never appeared in front of the camera but were observed on the field (supplementary material, Table 1). Therefore, we cannot exclude the consumption of treated seeds by these farmland species.

## Discussion

Considerable numbers of seeds were left on the soil surface after sowing, with an average of 7.14 uncovered seeds per square metre across all fields on day 0, without clusters. This represents 71,400 seeds per hectare, which would constitute nearly 1.3 million surface seeds for a field like Barnham. The seed density was higher on Barnham farm (9.7 seeds/m^2^) than on Hartfield farm (5.87 seeds/m^2^). Studies tend to show that the seed depth is the main driver of seed availability on the soil surface and that the variance that exists is largely due to soil condition (Pascual et al. 1999a; De Snoo and Luttik 2004). Therefore, the difference of surface seed density between farms could be due to the soil variation more than the use of plough and harrow before drilling. Overall, the surface seed density was higher on the headlands than on the centre for all fields (Fig. 2). Often, the drilling equipment is less effective at the end of the seed line (headlands) and, a small area, where headlands and centre field overlap, is often drilled twice (De Snoo and Luttik 2004; McGee et al. 2018; Roy et al. 2019; Lennon et al. 2020a).

The seed density significantly decreased with time (days post-sowing) on all fields and this decrease was the highest during the first 3 days post-sowing (Fig. 1). Our analysis showed a significant positive relationship between total bird density and the average surface seed density for Barnham field (Fig. 4). The difference existing between fields could be due to the relatively high number of birds observed on Barnham farm compared to Hartfield fields 1 and 2. On day 0, Barnham was visited by a big flock of corvids (carrion crow, rook, and jackdaw). Our results showed that birds consumed the pesticide-treated seeds left available on the soil surface quickly after being sown, so that most are consumed within about 4 days (Fig. 1). Furthermore, from the camera footage, we discovered that mammals (European badger, *Meles meles*; wood mouse, *Apodemus sylvaticus*; and European rabbit, *Oryctolagus cuniculus*) also participated in the removal of the treated seeds in Hartfield fields. Additionally, some studies have shown the importance of invertebrates, such as ground beetles (Carabidae), on seed predation (weed or crop; Brust and House 1988; Mullin et al. 2005; Talarico et al. 2016).

Out of the 18 bird species observed on the fields, 10 were also observed eating seed in front of the camera traps and 1 species—the feral pigeon—was seen exclusively in front of the camera traps. Species such as chaffinch, magpie, or European robin were exclusively found on boundaries, whereas woodlarks were only observed in front of camera traps placed in the centre of the field. All other species were observed in both locations. Because the headlands had a higher number of surface seeds, birds foraging on field boundaries might have a higher risk of exposure to harmful doses than birds feeding at the field centre (De Snoo and Luttik 2004). Interestingly, carrion crows seemed to keep seeds in their buccal cavity, with the throat visibly filling up during feeding in front of the camera, while the chaffinches were the only bird species observed de-husking seeds. Previous studies have shown that birds’ exposure could be considerably reduced by de-husking seeds but estimating an exact quantity is difficult (Edwards et al. 1998; Avery et al. 1997). Prosser and Hart (2005) suggested that smaller birds de-husk more than bigger birds, although small birds do not always de-husk seeds. This may be due to pressure to feed quickly because of risk of predation, competition for food, or space at the feeding site. Chaffinches were frequently present as a flock in front of the camera, which might explain why they spent more time feeding on the ground and de-husking seeds. Studies have also shown that an increased mean food intake was associated with birds foraging in groups, particularly for seed-eating bird species (Beauchamp 1998). Some seed-eating bird species were not observed in front of the cameras but were observed in the field (supplementary material, Table 1), which may potentially be due to those species being more neophobic, or because bigger species, such as corvids, pheasants, or partridges, monopolising the seeds. We suggest that camera traps are an excellent tool to determine the consumption rate of treated seeds by granivorous birds but that they should not be used in isolation to determine which species feed on treated seeds.

The extrapolation of the dose ingested for other pesticides used as seed treatment, on the base of a worst-case scenario and not considering the palatability differences that may exist between pesticides, showed that the neonicotinoid insecticide imidacloprid is the most likely to cause harm to birds feeding on crop treated seeds, due to its high toxicity (low LD50s, Table 2). The neonicotinoid insecticides clothianidin and thiamethoxam, the pyrethroid insecticide tefluthrin, and the anilide fungicide carboxin also showed a significant toxic potential. This was especially true for the feral pigeon and common chaffinch due to the large amount of seeds ingested per minute compared to their body weight.

Our study and the extrapolation we conducted suggest that if birds ingested seeds treated with imidacloprid at the same consumption rate as with fludioxonil, they would be likely to suffer some direct mortality. This is in accordance with previous studies, which have found that even a small number of imidacloprid-treated seeds could cause sublethal effects or even mortality to some bird species (Mineau and Palmer 2013; Goulson 2013; Gibbons et al. 2015; Eng et al. 2019).

While birds appear unlikely to receive an LD50 of the other pesticides, harmful sublethal effects can occur at much lower doses. For example, signs of toxicity in eared doves (*Zenaida auriculata*) appear with consumption of only 0.4g of imidacloprid-treated sorghum seeds, a tiny fraction of the daily intake (Addy-Orduna et al. 2019). In red munia (*Amandava amandava*), imidacloprid was shown to impair the hypothalamic-pituitary-thyroid and hypothalamic-pituitary-testicular axis leading to thyrotoxicity and testicular regression at only 0.25% or 0.5% of the LD50 (Pandey and Mohanty 2015; Mohanty et al. 2017; Pandey and Mohanty 2017). Eng et al. (2019) showed that consuming less than 5 imidacloprid-treated seeds were sufficient to have negative effects on migration and fat storing in white-crowned sparrows (*Zonotrichia leucophrys*). Both imidacloprid and clothianidin insecticides have been shown to create reproductive impairment and abnormal behaviour in male quails at much lower doses than their respective LD50s (Northern bobwhite quails (*Colinus virginianus*): Gibbons et al. 2015; Gobeli et al. 2017; Japanese quail (*Coturnix japonica*): Tokumoto et al. 2013).

While there has been much research on the effects of insecticides on birds, other pesticides have received little or no attention. Fungicides and herbicides tend to be less acutely toxic to non-target species than insecticides, but are applied in higher quantities than insecticides (Lopez-Antia et al. 2016; Tassin de Montaigu and Goulson 2020). A handful of studies have investigated the effects of seed treatment with fungicides on bird species (McGary et al. 2001; Grote et al. 2008; Satre et al. 2009; Köhler and Triebskorn 2013; Lopez-Antia et al. 2013, 2015, 2018; Pandey and Mohanty 2015; Gross et al. 2020; Ortiz-Santaliestra et al. 2020; Mateo et al. 2016). Thiram was found to reduce egg size, clutch size, number of fertile eggs, and brood size in red-legged partridge (Lopez-Antia et al. 2013, 2015; Pandey and Mohanty 2015). The fungicide difenoconazole also reduced egg size, number of fertile eggs, and the hatching rate in red-legged partridge (Lopez-Antia et al. 2013). The triazole fungicide flutriafol decreased the clutch size and number of fertile eggs and reduced by 50% the brood size in red-legged partridge (Lopez-Antia et al. 2018). Tebuconazole, another triazole fungicide, increased chick mortality in red-legged partridge when applied as a spray using field-realistic doses (Ortiz-Santaliestra et al. 2020). The dicarboximide fungicide vinclozolin was shown to impact behaviour and social interactions in dark-eyed junco (*Junco hyemalis*; Satre et al. 2009) and impair male reproductive behaviour in Japanese quail (McGary et al. 2001).

Mateo et al. (2016) found that red-breasted geese were potentially exposed to four fungicides (thiram, tebuconazole, difenoconazole, and fludioxonil) when feeding on germinated winter wheat seeds. By using daily food intake, they estimated the exposure levels of the birds and found that thiram and tebuconazole could represent a risk for geese. Gross et al. (2020) administered realistic quantities of fludioxonil-treated wheat seed to Japanese quails (low dose: 0.0328 mg/kg b.w, high dose: 0.0985 mg/kg b.w) and found that fludioxonil did not seem to bioaccumulate in tissues when dosed for 1 or 10 consecutive days. We were unable to find any studies specifically looking at sublethal impacts of the pyrethroid insecticide tefluthrin and the aniline fungicide carboxin on birds. Nonetheless, Millot et al. (2015) and Bro et al. (2016) both found traces of tefluthrin in grey partridge (*Perdix perdix*) adults and eggs, and Corcellas et al. (2017) found pyrethroid levels in 93% of wild bird eggs studied, demonstrating that exposure does occur in the wild. Other pyrethroid insecticides have sublethal neurotoxic effects on birds (Sánchez-Bayo 2012), showing behavioural effects in Japanese quail and potentially causing birth defects (David 1982) and inhibiting Japanese quails’ liver enzymes (Riviere et al. 1983). No publications studying effects of aniline fungicides on birds were found.

Our study focuses on short-term exposure in a single feeding bout or day. However, the wild birds observed are likely to feed for several days in numerous fields across the landscape, until the surface grain supply has been depleted. Winter wheat sowing is not synchronised across all fields of an area and is often sown from September to the end of October. Thus, birds could be exposed to pesticide-treated seeds for several weeks. The effects of such chronic exposure have been little investigated, although significant sublethal effects have been found in red munia after 30 days of exposure to 0.25% or 0.5% of the imidacloprid LD50 (Pandey and Mohanty 2015; Pandey and Mohanty 2017; Mohanty et al. 2017) and after 30 days of exposure to 1mg or 50mg of clothianidin per kilogramme of body weight in male quail (Tokumoto et al. 2013). Additionally, it is likely for birds to consume seeds coated or sprayed by different pesticides in adjacent fields and/or seeds treated with pesticide mixtures (Green et al. 2005; Köhler and Triebskorn 2013; Milner and Boyd 2017; Dudley et al. 2017; Stanton et al. 2018). Some interactions could occur between those several substances; these could diminish or enhance the toxic effects associated with a single active substance exposure (Larsen et al. 2003; Reffstrup et al. 2010).

A key assumption of our calculation of the doses of pesticides birds may receive from consuming treated seeds is that their palatability would be similar to those treated with fludioxonil. We are not aware of any trial to determine how different pesticide coatings affect seed palatability. Trials have been conducted to compare consumption of treated versus untreated seeds in captivity, and when given the choice birds sometimes show a preference for untreated seeds, depending on what the treatment is (Werner et al. 2010; Pascual et al. 1999b; Bennett and Prince 1981; Avery et al. 1993; Lopez-Antia et al. 2014). Consumption of treated seeds may drop over time as toxic effects begin to manifest, as, for example, occurring in eared doves fed imidacloprid-treated seeds (Addy-Orduna et al. 2019, Avery et al. 1994; Lopez-Antia et al. 2013; Millot et al. 2017; Botha et al. 2018). Conversely, the repellency of seeds coated with the fungicides thiram and carboxin or pyrethroid insecticides may diminish over time in the absence of alternative food and possibly even form addictive effects (David 1981; Kennedy and Connery 2008; Werner et al. 2010; Lopez-Antia et al. 2014).

Aside from palatability, other factors present in a natural environment such as hunger/starvation (Pascual et al. 1999c), predation risk (Avery et al. 1994), food unpredictability and accessibility (Lopez-Antia et al. 2014; Murton and Visozo 1963; Browns 1968), or competition (McKay et al. 1999) are all likely to influence consumption rates of different bird species in real-world situations. Complex though, all of these effects may be field evidence of bird fatality due to imidacloprid-treated and neonicotinoid-treated seed poisoning has been discovered, showing that wild birds do not always avoid eating treated seeds (Berny et al. 1999; Bro et al. 2004, 2010; Turaga et al. 2016; Millot et al. 2017; Ertl et al. 2018).

In conclusion, our study finds that large quantities of treated seed are left available for wildlife to consume after sowing of winter wheat and that these are consumed by a broad range of farmland bird species. When extrapolated to other pesticides used as seed treatment, this could lead to the ingestion of sufficient pesticide to induce sublethal and lethal effects, particularly following chronic exposure over multiple weeks. Clearly, further work is needed to examine how seed palatability is affected by different seed treatments and how this varies between bird species. Importantly, there is potential for harm from chemicals other than neonicotinoids, which until now have received the bulk of attention.

## Supplementary Information

High Resolution (TIFF 12685 kb)

## Data Availability

The datasets used and/or analysed during the current study are available from the corresponding author on reasonable request.
