# Supplementary figures and images for: Field evidence of UK wild bird exposure to fludioxonil and extrapolation to other pesticides used as seed treatments
Source: Environ Sci Pollut Res Int. 2021 Nov 15;29(15):22151–62. doi: 10.1007/s11356-021-17097-y (PMC8930954; doi:10.1007/s11356-021-17097-y)

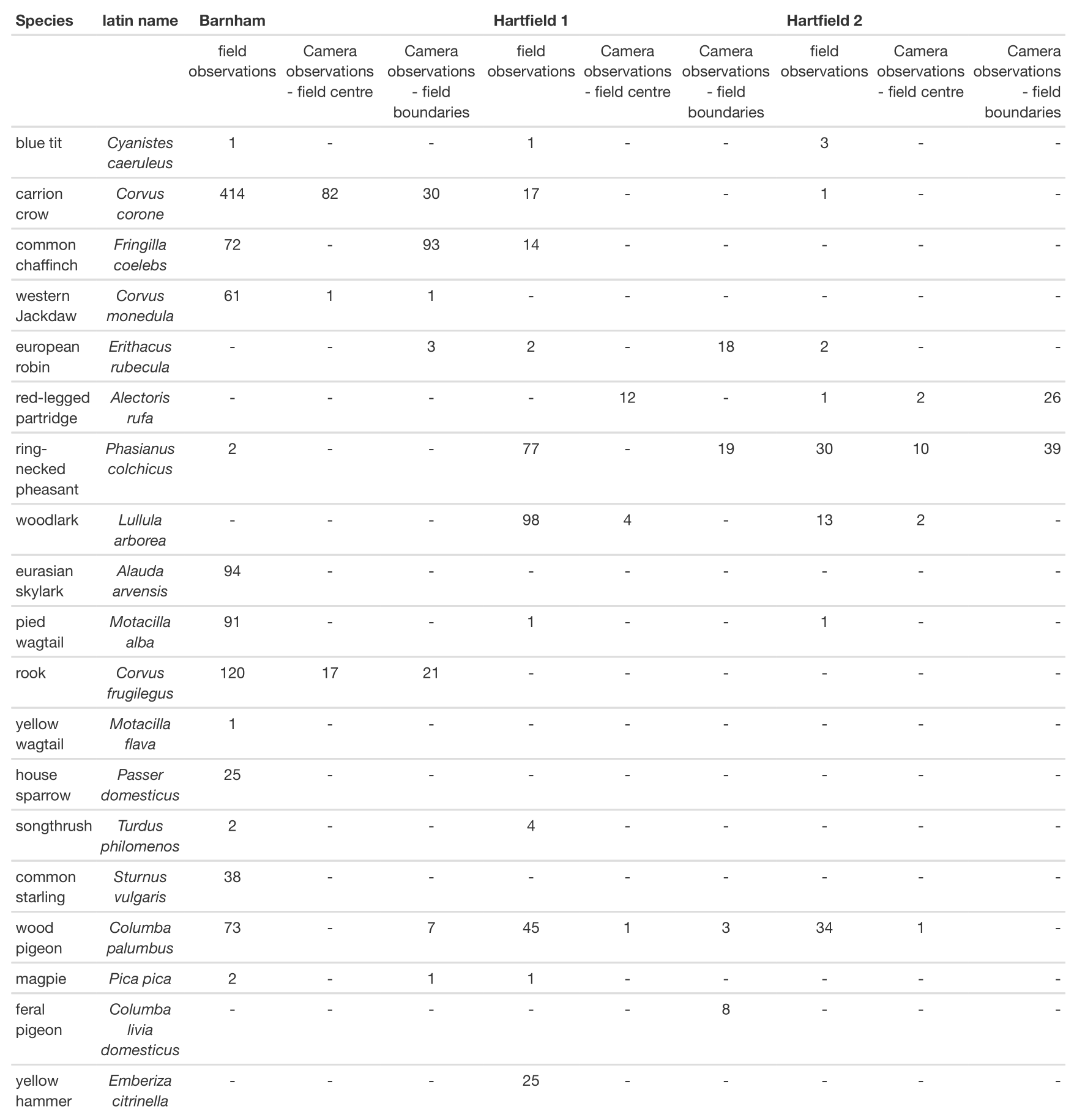

Supplement: Supplementary file 1 [file 11356_2021_17097_MOESM2_ESM.png]

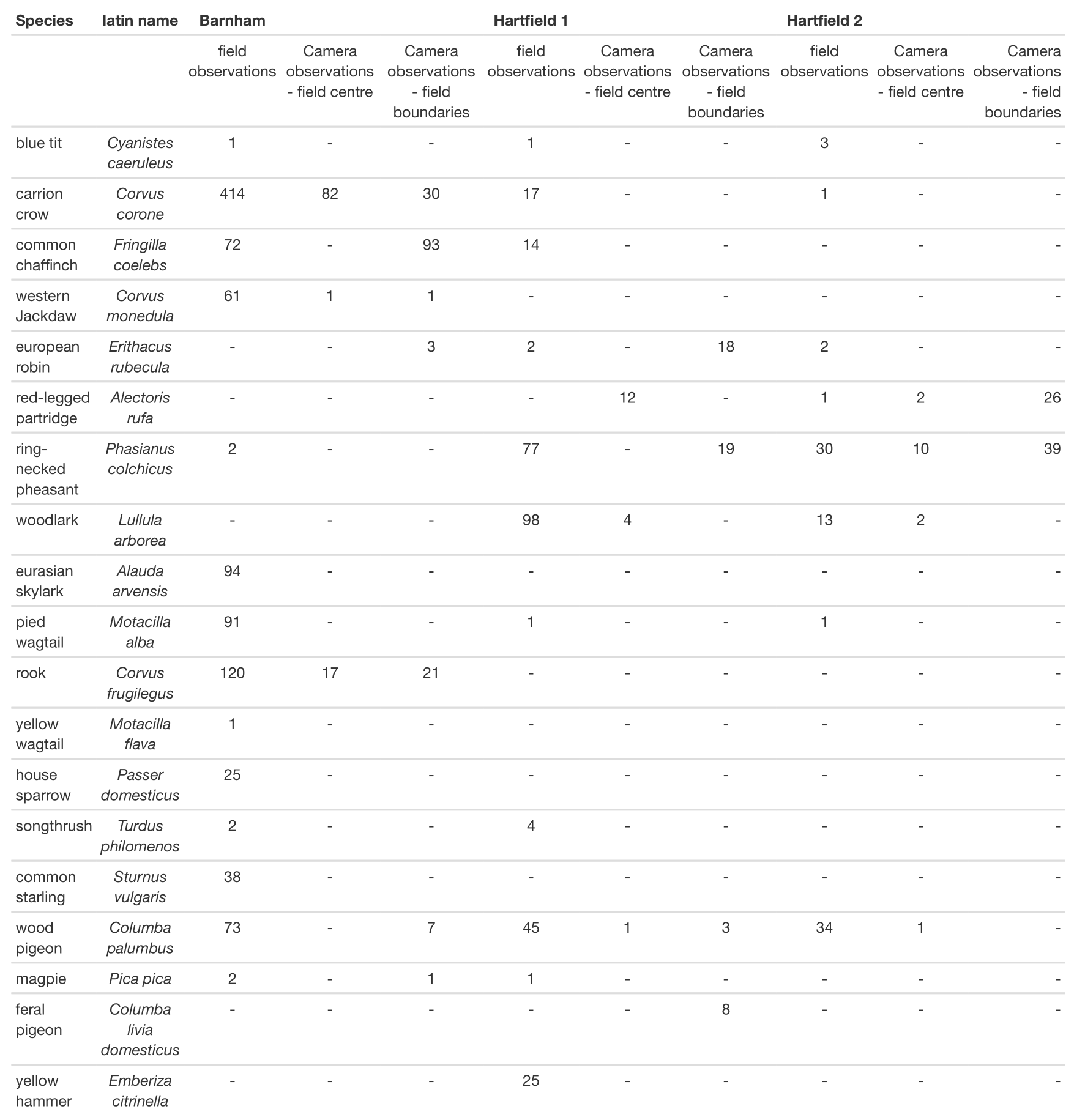

Supplement: Supplementary file 2 — High Resolution (TIFF 12685 kb) [file 11356_2021_17097_MOESM1_ESM.tiff]
